# Evaluation of sexual functional status and consistency of scales in patients with hypogonadotropic hypogonadism before and after testosterone replacement therapy: a single-center experience

**DOI:** 10.20945/2359-3997000000539

**Published:** 2022-12-01

**Authors:** Umit Aydogan, Yusuf Cetin Doganer, Cem Haymana, Umit Kaplan, Aydogan Aydogdu, Ibrahim Demirci, Coskun Meric, Yusuf Alper Sonmez

**Affiliations:** 1 University of Health Sciences Gulhane School of Medicine Department of Family Medicine Ankara Turkey University of Health Sciences, Gulhane School of Medicine, Department of Family Medicine, Ankara, Turkey; 2 University of Health Sciences Gulhane Training and Research Hospital Department of Endocrinology and Metabolism Ankara Turkey University of Health Sciences, Gulhane Training and Research Hospital, Department of Endocrinology and Metabolism, Ankara, Turkey; 3 International Medicana Hospital Department of Endocrinology and Metabolism Ankara Turkey International Medicana Hospital, Department of Endocrinology and Metabolism, Ankara, Turkey; 4 University of Health Sciences Gulhane School of Medicine Department of Endocrinology and Metabolism Ankara Turkey Department of Endocrinology and Metabolism, Gulhane School of Medicine, University of Health Sciences, Ankara, Turkey

**Keywords:** Hypogonadotropic hypogonadism, sexual functions, testosterone replacement therapy

## Abstract

**Objective:**

This study aimed to investigate the frequency of sexual dysfunction and the effect of short-term testosterone replacement therapy on sexual functions in congenital hypogonadism patients. Furthermore, we sought to reveal the consistency of the self-report scales used for the diagnosis of sexual dysfunction and the relationship between biochemical parameters.

**Materials and methods:**

The study was conducted on 47 young male patients aged above 18 years who were diagnosed with hypogonadotropic hypogonadism. Short (IIEF-5) and long (IIEF-15) forms of the International Index of Erectile Function and Arizona Sexual Experiences Scale (ASEX) were applied before treatment under the supervision of a physician. The patients’ blood pressure, height, and weight were measured, and their luteinizing hormone (LH), FSH, and total testosterone levels were recorded. Patients who started their treatments were called for a follow-up checkup after 6 months. Their blood pressure, height, and weight were measured by reapplying the ASEX, IIEF-5, and IIEF-15. In addition, their LH, FSH, and total testosterone levels in the biochemical tests were rerecorded.

**Results:**

In this study, the sexual dysfunction status of patients diagnosed with hypogonadotropic hypogonadism before and after treatment was evaluated using the ASEX, IIEF-15, and IIEF-5 scales. A decrease in sexual dysfunction was observed in all three scales after treatment compared with that before treatment. The IIEF-5 and IIEF-15 scales were found to be uncorrelated in terms of the pretreatment values but were correlated in terms of the post-treatment values. Although a correlation was observed between ASEX and IIEF-5 before treatment, no correlation was detected between ASEX and IIEF-15. After the treatment, ASEX was found to be correlated with both IIEF-5 and IIEF-15. The results of the scales indicated the correlation in all categories, except the pretreatment results of the IIEF-15 scale.

**Conclusion:**

The results of the current study demonstrated a significant improvement in the sexual function of hypogonadism patients undergoing short-term testosterone therapy. The ASEX, IIEF-5, and IIEF-15 scales used in the diagnosis and follow-up of sexual dysfunction were useful for evaluating sexual functions in hypogonadotropic hypogonadism patients.

## INTRODUCTION

Hypogonadism in young adults is a clinical condition characterized by insufficient testosterone production and/or impaired sperm production. Hypogonadotropic hypogonadism (secondary hypogonadism) refers to the inability to secrete gonadotropins (luteinizing hormone (LH) and/or FSH) due to an intrinsic or functional abnormality in the hypothalamus or pituitary gland ( 1 ). Although symptoms such as short stature, decreased self-confidence, depressed mood, difficulty or loss of concentration, forgetfulness, sleep disturbance, decreased parameters including muscle strength, mass, physical performance, working performance could be seen. The most significant symptoms are incomplete or delayed sexual development, loss of libido, and erectile dysfunction (ED) ( 2 ).

Sexual dysfunction in hypogonadism is one of the most important symptoms that can affect patients’ psychological state and impair their quality of life. Although laboratory tests are required for diagnosis, many physicians use reliable and valid self-report scales to assess the degree of sexual dysfunction and monitor the effectiveness of treatment. Many scales have been developed to evaluate sexual dysfunctions. These scales play a crucial role in the diagnosis, follow-up, and evaluation of the treatment outcome of sexual dysfunctions ( 3 ).

This study aimed to investigate the frequency of sexual dysfunction and the effect of short-term testosterone replacement therapy on sexual functions in congenital hypogonadism patients. Furthermore, it attempts to demonstrate the consistency of the self-report scales used to diagnose sexual dysfunction and the relationship between biochemical parameters.

## MATERIALS AND METHODS

### Study design and population

The study was conducted on 47 young male patients aged between 18 and 30 years who were diagnosed with congenital hypogonadotropic hypogonadism (CHH) between October 2012 and December 2016 at the Health Sciences University Gulhane Training and Research Hospital Endocrinology and Metabolic Diseases Clinic. Patients who were previously given testosterone or human chorionic gonadotropin (hCG) therapy and those with any chronic diseases or organ dysfunction were excluded. CHH was diagnosed based on a failure to undergo spontaneous puberty before the age of 18, which was confirmed by low total serum testosterone levels and normal or low gonadotropin levels. Pituitary hormones were evaluated in all patients to exclude panhypopituitarism. Pituitary or hypothalamic mass lesions were excluded by magnetic resonance imaging.

A questionnaire was given to the patients, which contained questions about age, educational status, marital status, habits, sociodemographic characteristics, and medical history. The patients filled out the questionnaires under the supervision of the responsible physician. In addition, IIEF-5, IIEF-15, and ASEX were applied before treatment also under the supervision of a physician.

The height, weight, and waist circumference (WC) of the patients and the control subjects were measured. WC was measured on the line between the iliac crest and the lower costal margin parallel to the ground after the subjects exhaled.

Patients who started their treatments were called for a follow-up checkup after 6 months. Their blood pressure, height, and weight were measured by reapplying ASEX, IIEF-5, and IIEF-15. In addition, their LH, FSH, and total testosterone levels in the biochemical tests were rerecorded.

### Instruments

#### Arizona Sexual Experiences Scale (ASEX):

This scale was developed by Mcgahuey and cols. in 2000 to appropriately evaluate the changes and disorders in the sexual functions of patients using psychotropic drugs. It is a Likert-type self-assessment scale with a score ranging from 1 to 6 for each question and consists of 5 questions. There are separate forms for men and women. In our study, only the male form was used, which contains questions regarding sexual drive, psychological arousal, erectile function (EF), orgasmic function (OF), and intercourse satisfaction (IS). High scores ranging from 5 to 30 indicate a sexual disorder. A total ASEX score of 19 or greater, any 1 item with a score of 5 or greater, or any 3 items with a score of 4 or greater have all been correlated with impaired sexual function ( 4 ). Soykan conducted the Turkish validity and reliability study on patients with end-stage renal disease ( 5 ).

#### IIEF (International Index of Erectile Function):

This scale was developed by Rosen and cols. in 1997 ( 6 ). Its Turkish translation as well as validity and reliability study were carried out by the Turkish Society of Andrology ( 7 ). It is widely used for evaluating the treatment results and ED. The studies on the reliability of the Turkish IIEF conducted by Bayraktar in 2017 were reviewed ( 8 ). IIEF-15 includes six questions regarding EF, three regarding IS, and two regarding OF, sexual desire, and general satisfaction. Contrary to ASEX, ED problems have been shown to increase as the scores obtained in IIEF-15 decrease.

Because the original scale was created to evaluate response to treatment in clinical studies, clinicians evaluated the need for a shorter scale for the diagnosis of ED. Thus, a five-question short variant (IIEF-5) was developed by Rosen and cols ( 9 ). The Turkish validity and reliability study was conducted by Turunç and cols. ( 10 ). Whereas IIEF-15 evaluates the last 4 weeks, IIEF-5 evaluates the last 6 months of their lives. Similar to IIEF-15, as the scores obtained in IIEF-5 decrease, ED problems increase.

IIEF-15 and IIEF-5 were systematically compiled by Neijenhuijs and cols. for their measurement characteristics ( 11 ).

### Testosterone replacement therapy

Sustanon ampoule^®^ (mixture containing 30-mg testosterone propionate, 60-mg testosterone phenylpropionate, 60-mg testosterone isocaproate, and 100-mg testosterone decanoate) was administered intramuscularly to the patients once every 3 weeks as testosterone replacement therapy.

### Laboratory measurements

In the diagnosis of hypogonadotropic hypogonadism, blood samples were taken intravenously from the antecubital region at 08:00 the next day after 1 night of fasting for biochemical analysis. Total testosterone, FSH, and LH blood samples were evaluated using the chemiluminescence method and the Beckman Coulter brand UniCel Dxl 800 model device. The cutoff value for testosterone was taken as 300 ng/dL; for FSH, 15 ng/dL; and for LH, 10 ng/dL. Blood samples were taken just before the first testosterone injection to evaluate the baseline parameters. The patients were followed up for 6 months, and then a final examination was conducted. The follow-up visits were arranged on days just before the next testosterone administration. Therefore, the time points for taking the blood samples were the same for all participants.

### Statistical analysis

Statistical evaluations were conducted by running the SPSS software (version 22.0; SPSS, INC., Chicago, IL, USA). The normal distribution of continuous variables was evaluated using the Kolmogorov-Smirnov test, histogram, and Shapiro-Wilk test. Descriptive statistics were expressed as mean ± standard deviation or median (minimum-maximum) for continuous variables and as a number of cases and percentage for categorical variables. Because the scale scores did not exhibit normal distribution; the Wilcoxon signed-rank test was employed for dependent pairwise group comparisons of continuous variables. McNemar test was used in the dependent group comparisons of categorical variables. Spearman’s correlation analysis was employed to evaluate the relationship between scale scores. *P* ≤ 0.05 was considered statistically significant.

### Ethics committee approval

The planning and implementation of the research were performed in accordance with scientific ethical principles and Declaration of Helsinki. Patients who wanted to participate in the research were informed that they could freely decide whether to participate or not, they could withdraw participation at any time, no invasive attempts specific to the study would be made, their personal information would not be shared, and the data obtained would be used only for scientific purposes. Written consent was obtained from all the participants. The local ethics committee approved the study protocol on 05 September 2012 (Number: 07).

## RESULTS

The mean age of the 47 patients included in the study was 21.07 ± 1.80 (19-28 years). Only 1 of them was married, and 33 (70.2%) received education for 8 years or less. Whereas the mean body mass index (BMI) of the patients upon admission to the hospital was 24.98 ± 4.39 kg/m^2^, it was 26.54 ± 4.71 kg/m^2^ at the 6th month after treatment ( *P* = 0.001). The median of the total testosterone values measured upon admission was 24.44 (1.00-178.30) ng/dL, and the median of the total testosterone values measured at the 6th month after the treatment was 97.00 (2.00-181.00) ng/dL ( *P* < 0.001) ( Table 1 ).

**Table 1:** Evaluation of some physical parameters and hormonal profiles of hypogonadotropic hypogonadism patients

	Pre-treatment Median (Min-Max)	Post-treatment (6-Month) Median (Min-Max)	*p* *
BMI (kg/m^2^) (Mean ± SD)	24.98 ± 4.39 (12.98-34.78)	26.54 ± 4.71 (16.98-36.57)	**0.001** **
Total testosterone (ng/dL)	24.44 (1.00-178.30)	97.00 (2.00-181.00)	**<0.001**
Waist circumference (cm)	89 (68-112)	94 (79-104)	**0.043**
FSH (ng/dL)	1.56 (0.03-84.38)	0.84 (0.01-61.27)	0.131
LH (ng/dL)	1.08 (0.01-39.70)	0.17 (0.04-35.17)	0.657

* Wilcoxon signed-rank test.

** Paired samples t-test.

Evaluation of the IIEF-5 and ASEX scale scores indicating the ED of the participants revealed that there were significant regressions in terms of ED according to the 6th-month scale scores before and after the treatment (8.00 *vs.* 15.00 [ *P* < 0.001] and 18.00 *vs.* 14.00 [ *P* < 0.001], respectively) ( Table 2 ). In the analysis of the IIEF-15 subgroup scores, significant increases in terms of functionality and satisfaction were observed in the pretreatment and 6th-month post-treatment scale scores in the areas of EF, OF, and IS (6.00 *vs.* 13.00 [ *P* < 0.001], 0.00 *vs.* 4.00 [ *P* < 0.001]), and 0.00 *vs.* 3.00 [ *P* < 0.001], respectively) ( Table 2 ).

**Table 2:** Evaluation of the pre-treatment and post-treatment IIEF-5 scale scores in hypogonadotropic hypogonadism patients

	IIEF-5		p *
	Pre-treatment Median (Min-Max)	Post-treatment (6-Month)(Min-Max)	
	8.00 (5-25)	15.00 (5-25)	**<0.001**
	ASEX		
	Pre-treatment Median (Min-Max)	Post-treatment (6-Month) Median (Min-Max)	
	18.00 (7-30)	14.00 (5-25)	**<0.001**
	IIEF		
	Pre-treatment Median (Min-Max)	Post-treatment (6-Month) Median (Min-Max)	
EF	6.00 (1-26)	13.00 (6-30)	**<0.001**
OF	0.00 (0-10)	4.00 (2-12)	**<0.001**
SD	5.00 (2-10)	6.00 (2-10)	0.362
IS	0.00 (0-9)	3.00 (3-13)	**<0.001**
OS	4.00 (2-9)	5.00 (2-10)	0.109

* Wilcoxon signed-rank test.

IIEF: International Index of Erectile Function; ASEX (Male form): Arizona Sexual Experiences Scale Male Form; EF: erectile function; OF: orgasmic function; SD: sexual desire; IS: intercourse satisfaction; OS: overall satisfaction.

**Figure 1:**
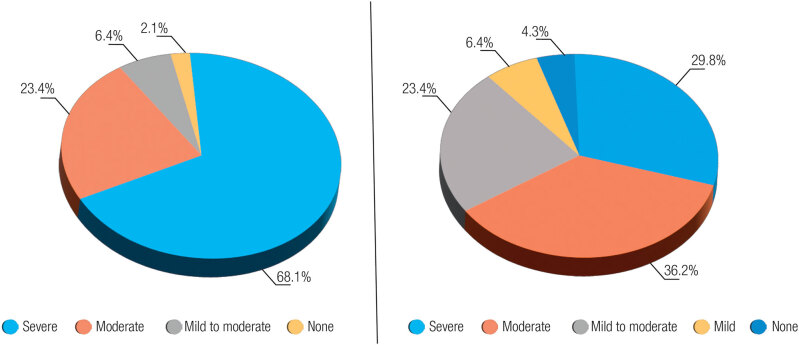
Evaluation of the IIEF-15 scale ED scores before and after treatment in hypogonadotropic hypogonadism patients.

**Figure 2:**
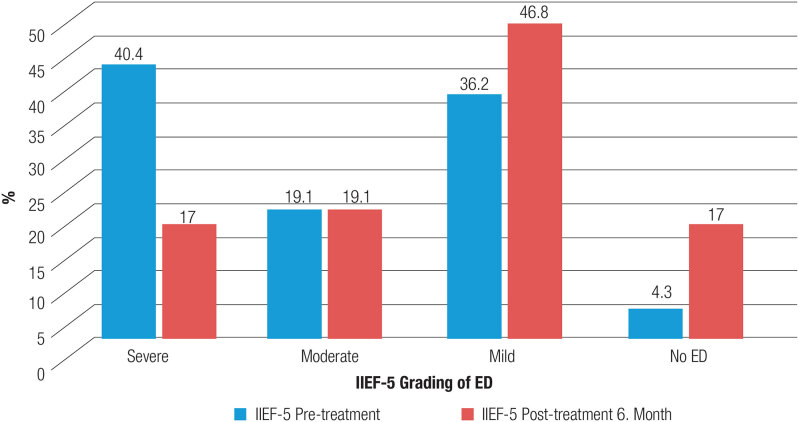
Evaluation of the IIEF-5 scale ED scores before and after treatment in hypogonadotropic hypogonadism patients.

**Figure 3:**
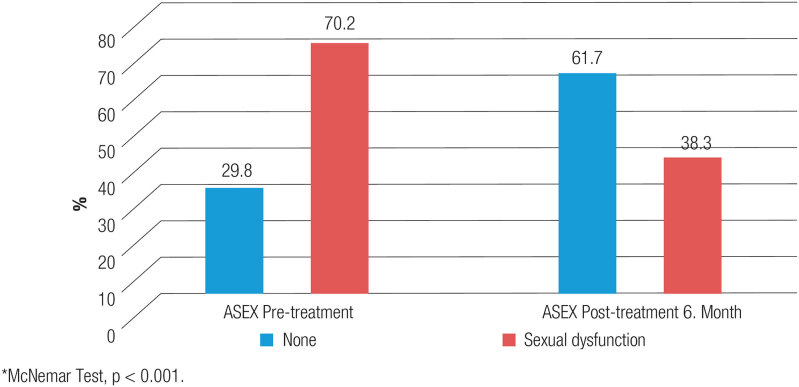
Assessment of the ASEX scale before and after treatment in hypogonadotropic hypogonadism patients.

According to the IIEF-15 scale score evaluation, whereas the rate of severe ED was 68.1% before treatment, it was 29.8% at 6 months post-treatment. Moreover, the rate of mild-to-moderate ED was 6.4% before treatment and 23.4% at 6 months post-treatment ( Figure 1 ). Contrarily, according to the IIEF-5 scale score evaluation, whereas the rate of severe ED was 40.4% before treatment, it decreased to 17% at 6 months post-treatment, whereas the proportion of those without ED symptoms increased from 4.3% to 17% ( Figure 2 ). According to the ASEX scale score evaluation results, the proportion of individuals with ED decreased from 70.2% before treatment to 38.3% at 6 months post-treatment ( *P* < 0.001) ( Figure 3 ).

No correlation was observed between the IIEF-5 scale scores measured upon admission before treatment and the IIEF subgroup scores and total testosterone levels in hypogonadotropic hypogonadism patients. On the other hand, a positive correlation was observed between the IIEF-5 scale scores measured at 6 months post-treatment and the scores of all the IIEF subgroups and total testosterone levels ( Table 3 ).

**Table 3:** Evaluation of the relationship between the IIEF-5 scale scores and IIEF scale sexual function scores and total testosterone in hypogonadotropic hypogonadism patients before treatment and 6 months after treatment

		IIEF-EF Pre-treatment	IIEF-OF Pre-treatment	IIEF-SD Pre-treatment	IIEF-IS Pre-treatment	IIEF-OS Pre-treatment	TOTAL TESTOSTERONE Pre-treatment
IIEF-5	p	0.758	0.456	0.837	0.998	0.998	0.241
Pre-treatment	r	0.046	0.111	0.031	0.000	0.000	0.174
		**IIEF-EF** **Post-treatment (6-Month)**	**IIEF-OF** **Post-treatment (6-Month)**	**IIEF-SD** **Post-treatment (6-Month)**	**IIEF-IS** **Post-treatment (6-Month)**	**IIEF-OS** **Post-treatment (6-Month)**	**TOTAL TESTOSTERONE** **Post-treatment (6-Month)**
IIEF-5- 6 – Month	p	**0.001**	**0.011**	**0.000**	**0.001**	**0.000**	**0.027**
	r	0.478	0.367	0.568	0.465	0.760	0.324

IIEF: International Index of Erectile Function; EF: erectile function; OF: orgasmic function; SD: sexual desire; IS: intercourse satisfaction; OS: overall satisfaction.

A significant negative correlation was observed between the ASEX scores upon admission before treatment and the IIEF-5 scale scores of hypogonadotropic hypogonadism patients ( *P* = 0.046; r = −0.293). However, there was no correlation between the IIEF subgroup scores and total testosterone levels. A significant negative correlation was measured between the ASEX scores at 6 months post-treatment and the IIEF-5 scale scores of the hypogonadotropic hypogonadism patients ( *P* = 0.003; r = −0.421). Furthermore, a negative correlation was observed in the IIEF subgroup scores in other than OF. No correlation was found in the total testosterone level of hypogonadotropic hypogonadism patients ( Table 4 ).

**Table 4:** Evaluation of the relationship between the pre-treatment and post-treatment 6th-month ASEX scores and other sexual function scale scores and total testosterone in hypogonadotropic hypogonadism patients

		IIEF-5 Pre-treatment	IIEF-EF Pre-treatment	IIEF-OF Pre-treatment	IIEF-SD Pre-treatment	IIEF-IS Pre-treatment	IIEF-OS Pre-treatment	TOTAL TESTOSTERONE Pre-treatment
ASEX	p	**0.046**	0.791	0.849	0.882	0.609	0.794	0.609
Pre-treatment	r	−0.293	−0.040	0.028	0.022	0.077	0.039	−0.076
		**IIEF-5 Post-treatment (6-Month)**	**IIEF-EF Post-treatment (6-Month)**	**IIEF-OF Post-treatment (6-Month)**	**IIEF-SD Post-treatment (6-Month)**	**IIEF-IS Post-treatment (6-Month)**	**IIEF-OSPost-treatment (6-Month)**	**TOTAL TESTOSTERONE Post-treatment (6-Month)**
ASEX	p	**0.003**	**0.010**	0.129	**0.000**	**0.001**	**0.000**	0.068
Post-treatment (6-Month)	r	−0.421	−0.371	−0.224	−0.575	−0.473	−0.542	−0.269

IIEF: International Index of Erectile Function; ASEX (Male form): Arizona Sexual Experiences Scale Male Form; EF: erectile function; OF: orgasmic function; SD: sexual desire; IS: intercourse satisfaction; OS: overall satisfaction.

## DISCUSSION

In our study, the pre- and post-treatment sexual dysfunction status of the patients, who were diagnosed with hypogonadotropic hypogonadism and treated for 6 months, was evaluated using the ASEX, IIEF-15, and IIEF-5 scales. Sexual dysfunction was found to decrease in all three scales after treatment compared with that before treatment. Based on the correlation between the results of the IIEF-5 and IIEF-15 scales, no correlation was observed in the pretreatment values, but the post-treatment values were found to be correlated. Furthermore, there was a correlation between ASEX and IIEF-5 but none between ASEX and IIEF-15 before treatment. After the treatment, ASEX was found to be correlated with both the IIEF-5 and IIEF-15 results. The results of the scales demonstrated correlation in all other categories, except the pretreatment results of the IIEF-15 scale.

One of the critical problems in hypogonadism patients is infertility due to impaired sexual functions. This clinical condition not only prevents an individual from having children but also impairs the quality of life. It is considered as a stress factor that can lead to sexual incompatibility between spouses ( 12 ). In our study, almost all the participants were not evaluated for infertility because they were not married. On the other hand, infertility problems are presumed to decrease depending on the improvement in the patients’ sexual functions due to the treatment, which will positively affect their quality of daily life, mood, and well-being. Testosterone replacement therapy affects patients’ libido, sexual thoughts, and sexual desire ( 13 , 14 ). In addition, it increases the frequency and duration of penile erections and improves EF ( 15 , 16 ). Various methods can follow the clinical results of the treatment given to hypogonadotropic hypogonadism patients. Different scales and questionnaires have been developed to monitor sexual problems. In our study, three scales, namely, ASEX, IIEF-15, and IIEF-5, were used to monitor the sexual functions of the study participants. In all three different scales, sexual problems were found to decrease statistically significantly after treatment than before treatment, and the results of our study are in agreement with those of other studies.

Testosterone replacement therapy improves sexual functions, increases libido, and promotes affirmative mood in hypogonadism patients ( 12 , 17 , 18 ). Studies are pointing out the results of using different methods of testosterone in the treatment of hypogonadism patients. Wang and cols. demonstrated that the testosterone gel form improved patients’ sexual functions and well-being ( 19 ). Jockenhövel and cols. investigated the effects of different modalities in testosterone replacement therapy on sexual functions. Although the results were inconsistent, sexual function was found to increase in all treatments examined ( 20 ). A study conducted by Aydogan and cols., in which they investigated the effects of the treatments given to hypogonadotropic hypogonadism patients, reported that compared with the control group, hypogonadism patients experienced increased sexual dysfunction, anxiety, and depression and had a lower quality of life. Improvements were observed in the same parameters after 6 months of testosterone replacement therapy ( 21 ). Another study conducted by Aydogan and cols. reported an increase in muscle mass and aerobic capacity after testosterone replacement therapy ( 22 ). In the study by Yassin and cols., which aimed to demonstrate the effect of testosterone replacement therapy on sexual functions in late-onset hypogonadism, the IIEF scale was used to evaluate sexual functions, and an increase of approximately 50% in sexual functions was observed after treatment than before treatment ( 23 ). In our study, the patients were treated with Sustanon ampoule^®^ (a mixture containing 30-mg testosterone propionate, 60-mg testosterone phenylpropionate, 60-mg testosterone isocaproate, and 100-mg testosterone decanoate) once every 3 weeks for 6 months. An increase in the testosterone levels measured in the follow-up checkups after the treatment and an improvement in sexual functions were observed in all the three scales. The results of our study are in agreement with those of other studies.

In addition to the scales for the follow-up of sexual functions, there are various studies that used biochemical parameters. In the study conducted by Sansone and cols. on hypogonadism patients, sexual functions were evaluated using IIEF and Aging Male’s Symptoms (AMS) questionnaire, and the relationship between the results and dihydrotestosterone was investigated. According to the study results, the dihydrotestosterone level was associated with hypogonadism symptoms in hypogonadism patients ( 24 ). A study conducted by Rosen and cols., which aimed to show the effect of TRT on the quality of life and sexual functions, reported that TRT increases the quality of life and improves the sexual functions of patients. The IIEF and AMS scales gave similar results ( 25 ). In the study conducted by Liang and cols., in which they examined the relationship between hypogonadism symptoms and biochemical parameters in aging men, the IIEF-5 and AMS scales were used, and a significant correlation was observed between LH and sex hormone binding globulin and sexual functions ( 26 ). In our study, the relationship between total testosterone and sexual functions was investigated, and no correlation was observed between the ASEX, IIEF, and IIEF-5 results, except for the IIEF-5 results after treatment. The results of our study indicated that total testosterone levels are not a suitable parameter in the follow-up of sexual functions.

There are various scales used to evaluate sexual functions. In a study by Öncel and cols. investigating the change in sexual functions following bariatric surgery, ASEX and IIEF-5 were used together. Both tests gave similar results in the preoperative and postoperative evaluations ( 27 ). Nunes and cols. investigated the effects of lodenafil and placebo treatment on sexual dysfunction in patients with schizophrenia, and ASEX and IIEF-15 were used to evaluate sexual functions. The results of ASEX and IIEF-15 were found to be similar ( 28 ). In parallel, pairwise comparisons of the scales were performed in various pathologies, and the scales provided similar results ( 29 – 32 ). However, in our literature review, we did not find any study comparing the results of ASEX, IIEF-15, and IIEF-5 in the evaluation of sexual functions among hypogonadotropic hypogonadism patients. Our study demonstrated a significant positive correlation between the IIEF-15 and IIEF-5 scales in the post-treatment values. Contrarily, no statistically significant correlation was observed in the pretreatment values. Based on the pre- and post-treatment results of the ASEX, IIEF-15, and IIEF-5 scales, a statistically significant negative result was obtained between the other results, except for the pretreatment values of ASEX and IIEF-15. As the scores in the ASEX increase, sexual functions deteriorate; contrarily, as the scores obtained in IIEF and IIEF-5 decrease, sexual functions deteriorate. Therefore, the negative correlation between the ASEX and IIEF scale results indicated that the results of the scales are similar. The lack of correlation between IIEF-15 and other scales in the pretreatment results could be due to the fact that there were 3 separate subgroups of similar questions in the IIEF-15 scale. An increase in the number of questions could negatively affect the patients’ compliance during the scale application. In addition, the Turkish validity and reliability study of IIEF conducted by Bayraktar and cols. reported that some comprehension problems required additional verification in the Turkish IIEF. However, these problems were resolved with the validity study. It has also been stated that IIEF is less reliable because of these comprehension problems.

Furthermore, it was stated that comprehension problems could be resolved with the help of physicians in the reported patient groups, and as a result, IIEF could be made reliable in all patient groups ( 8 ). Other studies conducted by Bayraktar and Altun demonstrated that the guidance of physicians helps eliminate the comprehension problem in IIEF ( 33 , 34 ). Therefore, the inconsistency in pretreatment IIEF-15 scores is considered to result from the inadequate understanding of the questions by the patients before the treatment. When all the results are evaluated, providing physician support to patients while applying IIEF-15 is appropriate. All scales can be used interchangeably to evaluate sexual functions in hypogonadotropic hypogonadism patients.

## Limitations

The most important limitation of this study is that despite its prospective design, it could not be evaluated as a randomized controlled trial due to the lack of a control group. Second, three scales with similar questions used in the current study may have negatively affected the compliance of the patients in the application of the scales. Furthermore, the pretreatment results of the IIEF-15 scale did not correlate with those of the other scales that support this situation. Our study evaluated the changes in sexual functions after testosterone replacement therapy at 6 months after treatment. Finally, a longer follow-up duration may provide more detailed information about the long-term effectiveness of the given treatment and the observation of possible side effects.

In conclusion, although hypogonadotropic hypogonadism is not very common in society, it negatively affects the sexual function, marriage, family, fertility, and sexual health of young adult men. Our study also demonstrated improvements in sexual dysfunctions after treatment with the application of correct treatment protocols. Early diagnosis and treatment of hypogonadotropic hypogonadism patients are essential for public health. Thus, adolescents and young adult patients, whom we encounter at every opportunity in healthcare delivery, should be careful in terms of sexual dysfunction while taking history. In addition, it is considered that the biopsychosocial approach as family physicians will be of vital importance in the detection and treatment of hypogonadism patients.

This study demonstrated a significant improvement in the sexual function of hypogonadism patients with short-term testosterone therapy. All three of the ASEX, IIEF-5, and IIEF-15 scales used to diagnose, and follow-up sexual dysfunction are functional scales evaluating sexual functions in hypogonadotropic hypogonadism patients. Although the IIEF-15 scale can give more detailed results due to many questions, it may give different results because the scale takes longer to apply in terms of time and difficulties in patient compliance. Due to the aforementioned reasons, it is considered that IIEF-5 may be used instead of IIEF-15.
